# Workplace violence by specialty among Peruvian medical residents

**DOI:** 10.1371/journal.pone.0207769

**Published:** 2018-11-29

**Authors:** Wendy Nieto-Gutierrez, Carlos J. Toro-Huamanchumo, Alvaro Taype-Rondan, Raúl Timaná-Ruiz, Carlos Alva Diaz, David Jumpa-Armas, Seimer Escobedo-Palza, CONAREME Consejo Nacional de Residentado Médico

**Affiliations:** 1 Facultad de Medicina Humana, Universidad de San Martín de Porres, Lima, Peru; 2 Unidad de Investigación para la Generación y Síntesis de Evidencias en Salud, Universidad San Ignacio de Loyola, Lima, Peru; 3 Association for the Study of Medical Education (ASME), Edinburgh, United Kingdom; 4 Instituto de Evaluación de Tecnologías en Salud en Investigación, EsSalud, Lima, Peru; 5 Red de Eficacia Clínica y Sanitaria (REDECS), Lima, Peru; 6 International Society For Pharmacoeconomics and Outcomes Research (ISPOR) capítulo Perú, Peru; 7 Universidad Científica del sur, Lima, Peru; 8 IBT Health, Hospital Guillermo Kaelin de la Fuente, Lima, Peru; 9 Sociedad Peruana de Administración en Salud, Lima, Peru; University of the West Indies at Saint Augustine, TRINIDAD AND TOBAGO

## Abstract

**Objective:**

To determine the prevalence of workplace violence among Peruvian medical residents and to evaluate the association between medical specialty and workplace violence per type of aggressor.

**Methods:**

This was a cross-sectional secondary analysis that used data from the Peruvian Medical Residents National Survey 2016 (ENMERE-2016). The outcome of interest was workplace violence, including physical and verbal violence, which were categorized according to the perpetrator of violence (patients/relatives and worker-to-worker). Primary exposure was the medical specialty, categorized as clinical, surgical, and other specialties. To evaluate the associations of interest, we estimated adjusted prevalence ratios (PR) with their respective 95% confidence intervals (95% CI) using Poisson regression models with robust variances.

**Results:**

A total of 1054 Peruvian medical residents were evaluated. The mean age was 32.6 years and 42.3% were female. Overall 73.4% reported having suffered of workplace violence sometime during the residency, 34.4% reported violence from patients/relatives, and 61.1% reported worker-to-worker violence. Compared with clinical residents, surgical residents had a lower prevalence of violence from patients/relatives (PR: 0.71; 95% CI: 0.59–0.87), but a higher prevalence of worker-to-worker violence (PR: 1.11, 95% CI: 1.01–1.23).

**Conclusion:**

Nearly three quarters of medical residents reported having suffered workplace violence sometime during their residency. Compared with clinical residents, surgical residents had lower rates of violence from patients/relatives, but higher rates of worker-to-worker violence; while residents from non-clinical and non-surgical specialties had a lower prevalence of both types of violence.

## Introduction

Workplace violence among physicians can be defined as any situation in which physicians are humiliated, threatened, or injured at work or on duty [1]. It has become a concern worldwide [2, 3] due to its high rates [4] and its impact on the costs of health services, the user dissatisfaction [5], and on physicians’ mental health, job performance, empathy with their patients, and quality of provided services [5].

Medical residents are physicians training to become specialists, who could have a higher risk of suffering from workplace violence. Since residents tend to spend more time with patients and their relatives than other physicians, they are potentially at a higher risk of suffering workplace violence perpetrated by their own patients [3]. On the other hand, due to their in-training status, residents may be prone to suffer workplace violence perpetrated by other health professionals such as their senior residents, attending physicians, nursing staff, among others [5, 6].

The issue of violence among medical residents has been reported in previous studies performed in United States [3, 7–11], Asia [12–15], and some Latin American countries: Mexico [16], Uruguay [17, 18], and Argentina [19]. However, most of these studies have been performed on residents of a specific medical specialty or on a specific health facility.

Workplace violence among medical residents could be higher in certain groups. Namely, specialties with higher contact with patients could have higher violence rates [4]. A few studies have evaluated the association between specialty and workplace violence in residents. A study in Turkey found that surgical residents were more prone to workplace violence than their clinical counterparts [14], Likewise a study in Argentina found that surgical residents had higher violence rates than other specialties [19].

Although some studies have evaluated workplace violence among physicians in Peru [20–23], none have evaluated it among residents. Thus, this study was aimed to determine the prevalence of workplace violence among Peruvian medical residents and to evaluate the association between medical specialty and workplace violence by type of aggressor.

## Methods

### Study design

This was a cross-sectional secondary analysis that used data from the Peruvian Medical Residents National Survey-2016 (*Encuesta Nacional de Médicos Residentes*-2016, ENMERE-2016), a voluntary country-level survey performed by the National Committee of Peruvian Medical Residency (*Consejo Nacional de Residentado Médico*, CONAREME).

CONAREME is the institution in charge of regulating residency training in medical schools and proposing rules and quality standards for the medical residency in Peru. This national committee is constituted by representatives from all the 23 medical schools that train residents in Peru [24].

In Peru, physicians perform their residency in a health institution belonging to one of the four health systems: 1) the Ministry of Health (MINSA), responsible for providing health services to the population living in conditions of poverty and extreme poverty, most of the Peruvian health institutions belong to MINSA [25]; 2) the Social Security (EsSalud), responsible for providing health services to state workers and their families, and funded by the Ministry of Labor; 3) the armed forces and police health system, responsible for providing health services to the armed forces and police, and funded by the corresponding ministries; or 4) the private sector, financed by its users [26].

### Study population

We included all medical residents who answered the online ENMERE-2016 questionnaire in our analyses, and excluded those who did not complete the variables of interest.

### Procedures

The questionnaire used for the ENMERE-2016 was elaborated by CONAREME, and was validated through focus groups, with medical residents, and expert opinion, which were identified as researchers who had previously published on the subject by Peruvian medical residents and Peruvian researchers in medical education. During June 2016, this questionnaire was placed online in a virtual platform in the CONAREME webpage. Medical residents were able to fill out the survey using their national identification number.

During June 2016, CONAREME contacted to the Peruvian residents to answer the survey, via residents’ personal emails, CONAREME’s Facebook, and Peruvian newspapers. The participation of the residents was voluntary, and their answers were stored in the CONAREME virtual platform database. The residents’ names and national identification numbers were not included in the final database.

### Outcome: Workplace violence

Our main outcomes were three: 1) workplace violence, 2) workplace violence from patients/relatives, and 3) worker-to-worker workplace violence.

Workplace violence was defined as having suffered either physical or verbal violence. To define these variables, we used the International Labor Office’s (ILO) definitions. Physical violence was defined as “the use of physical force against another person or group, that results in physical, sexual or psychological harm, includes beating, kicking, slapping, stabbing, shooting, pushing, biting, pinching, among others”. Verbal violence was defined a “intentional use of power, including threat of physical force, against another person or group, that can result in harm to physical, mental, spiritual, moral or social development”, including threats (defined as the promise of use of power or physical force towards an individual or group) and insults (defined as aggression against another person through the use of language, including insults, teasing, humiliations, and others) [27].

Workplace violence from patients/relatives was defined as having suffered workplace violence anytime during their residence and reported that perpetrators were patients or their relatives.

Worker-to-worker workplace violence was defined as having suffered workplace violence anytime during their residence and reported that perpetrators were any of the following: attending physician, senior resident, other residents, non-medical health professional, or other institution staff.

### Exposure: Medical specialty

Primary exposure was medical specialty, categorized by group of specialties: clinical specialties (specialties that focus on the diagnosis and non-surgical treatment of disease), surgical specialties (specialties focus on manually surgical techniques to treat disease), and other specialties (non-clinical, non-surgical specialties, such as radiology, pathology, legal medicine, etc.), as detailed in the Table 1 [28].

**Table 1:** Characteristics of Peruvian residents and residents included in the study.

Variable	Total of residents in Peru	Residents included in the analysis N	Residents included in the analysis %	Chi2 p
**University**				<0.001
Public university of Lima	2,628	-307	11.7%	
Private university of Lima	2,551	370	14.5%	
Public university not Lima	1,546	233	15.1%	
Private university not Lima	668	144	21.6%	
**Health institution**	** **	** **	** **	0.032
MINSA	4,573	696	15.2%	
EsSalud	2,208	281	12.7%	
Others	612	77	12.6%	
**Specialty**	** **	** **	** **	0.015
Clinical	3,704	521	14.1%	
Surgical	2,786	372	13.4%	
Other	903	161	17.8%	
**Year of residency**				<0.001
First year	2,713	432	15.9%	
Second year	2,642	287	10.9%	
Third year or higher	2,038	335	16.4%	

In addition, some clinical specialties (pediatrics, internal medicine, family and community medicine, and other clinical specialties) and some surgical specialties (general surgery, gynecology, and other surgical specialties) were evaluated separately.

### Other variables

Other demographic variables evaluated were: sex (male or female), age (in tertiles), marital status (single or married/cohabitant), and migration status (yes or no). Migration was defined as be performing the medical residency in a different province than where medical undergraduate studies were performed.

Other variables were: year of residency (first year, second year, or third year or higher), university responsible of the residency (public university of Lima, private university of Lima, public university of another city, and private university of another city), health system of the institution (MINSA, EsSalud, armed forces/police, or private), hours worked per day (in tertiles: <10 hours, 10 to 12 hours and >12 hours) and city where the residency is undertaken.

### Statistical analysis

Descriptive data were analyzed using means ± standard deviations and absolute and relative frequencies. We used the Chi-square test for associations between the population characteristics with type of violence.

To evaluate the associations of interest, we estimated adjusted prevalence ratios (PR) with their respective 95% confidence intervals (95% CI) using Poisson regression models with robust variances. These regressions were adjusted by sex, age, university responsible of the residency, city where the residency is undertaken, migration, year of residency, health system of the institution and hours worked per day. All statistical analyses were performed using Stata 14.0.

### Ethics

This study was reviewed and approved by the Institutional Ethics Review Board of the Hospital San Bartolome (RCEI-40 / Exp. N° 01651–17), Lima, Peru. For the use and analysis of the database of ENMERE-2016, the authors requested the respective permission to CONAREME. The ENMERE survey was voluntary. Confidentiality was guaranteed through the use of individual codes for data analysis.

### Availability of data and materials

The data of the ENMERE-2016 (Peruvian Medical Residents National Survey-2016) belongs to the National Committee of Peruvian Medical Residency. These are third party data and others would be able to access these data in the same manner as the authors. Those who were interested at the access of the database should request it to CONAREME (http://www.conareme.org.pe/web/).

## Results

### Characteristics of the population

During June 2016, 7393 physicians were performing a medical residency program in Peru according to CONAREME records, from which 1269 completed the ENMERE-2016. Data from 215 participants was subsequently eliminated for not having the variables of interest. Finally, data from 1054 residents (14.3% of the total) were analyzed. The percentage of respondents was higher among those from private universities in provinces, MINSA institutions' residents, non-clinical and surgical specialties, and senior residents (Table 1).

The mean age was 32.6 ± 5.4 years, 446 (42.3%) were female, 404 (38.3%) were married or coinhabiting, 677 (64.2%) were performing their residency in Lima, 696 (66.0%) belonged to a MINSA hospital, and 432 (41.0%) were first-year residents. Residents were performing their residency in 13 Peruvian cities: 677 (64.2%) in Lima, 115 (10.9%) in Trujillo, and 80 (7.6%) in Arequipa. Furthermore, 521 (49.4%) were clinical residents, 372 (35.3%) surgical residents, and 161 (15.3%) were residents of other specialties (Table 2).

**Table 2:** Characteristics of the population according to having suffered workplace violence (n = 1054).

Variables	Total	No workplace violence N = 280.	Workplace violence N = 774	Chi2 p
**Sex**				0.231
Male	608 (57.7)	170 (28.0)	438 (72.0)	
Female	446 (42.3)	110 (24.7)	336 (75.3)	
**Age**				0.356
20–29 years	364 (34.5)	106 (29.1)	258 (70.9)	
30–34 years	391 (37.1)	96 (24.6)	295 (75.4)	
35–54 years	299 (28.4)	78 (26.1)	221 (73.9)	
**Migration**				0.739
No	616 (58.4)	166 (26.9)	450 (73.1)	
Yes	438 (41.6)	114 (26.0)	324 (74.0)	
**Year of residency**				0.014
First year	432 (41.0)	135 (31.3)	297 (68.8)	
Second year	287 (27.2)	64 (22.3)	223 (77.7)	
Third year or higher	335 (31.8)	81 (24.2)	254 (75.8)	
**University**				0.219
Public university of Lima	307 (29.1)	76 (24.8)	231 (75.2)	
Private university of Lima	370 (35.1)	99 (26.8)	271 (73.2)	
Public university not Lima	233 (22.1)	57 (24.5)	176 (75.5)	
Private university not Lima	144 (13.7)	48 (33.3)	96 (66.7)	
**Health Institution**				0.116
MINSA	696 (66.0)	191 (27.4)	505 (72.6)	
EsSalud	281 (26.7)	66 (23.5)	215 (76.5)	
Armed forces/pólice	55 (5.2)	13 (23.6)	42 (76.4)	
Private	22 (2.1)	10 (45.5)	12 (54.5)	
**Specialty group**				<0.001
Clinical	521 (49.4)	120 (23.0)	401 (77.0)	
Surgical	372 (35.3)	89 (23.9)	283 (76.1)	
Other	161 (15.3)	71 (44.1)	90 (55.9)	
**Hours worked**				0.055
< 10 hours	423 (42.6)	124 (29.3)	299 (70.7)	
10–12 hours	485 (48.8)	122 (25.2)	363 (74.8)	
> 12 hours	86 (8. 6)	15 (17.4)	71 (82.6)	

### Overall workplace violence

Workplace violence from any aggressor was reported in 774 (73.4%) residents, workplace violence from patients/relatives was reported in 363 (34.4%), and worker-to-worker violence was reported in 644 (61.1%). Perpetrators of physical and verbal violence are detailed in Table 3.

**Table 3:** Physical and verbal violence according to aggressor (n = 1054).

Type of violence and perpetrators	Physical violence N (%)	Verbal violence N (%)	Total N (%)
Patients/Relatives	94 (8.9)	343 (32.5)	363 (34.4)
Attending physician	58 (5.5)	472 (44.8)	479 (45.5)
Senior resident	27 (2.6)	262 (24.9)	263 (25.0)
Younger or same-year resident	7 (0.7)	61 (5.8)	62 (5.9)
Non-medical health professional (Medical interns, nurses, technologists etc.)	16 (1.5)	120 (11.4)	128 (12.1)
Other institution staff (Administrative, security, etc.)	19 (1.8)	122 (11.6)	128 (12.1)

### Workplace Violence according to medical specialty

Prevalence of workplace violence was 77.0% in clinical residents, 76.1% in surgical residents, and 55.9% in residents of other specialties. Compared with clinical residents, this prevalence was similar in surgical residents (PR: 0.99; 95% CI: 0.91–1.07), but lower in the group of other specialties (PR: 0.75; 95% CI: 0.64–0.87).

Prevalence of workplace violence from patients/relatives was 42% in clinical residents, 29.3% in surgical residents, and 21.7% in residents of other specialties. Compared with clinical, this prevalence was lower in surgical residents (PR: 0.71, 95% CI: 0.59–0.87) and in the group of other specialties (PR: 0.48, 95% CI: 0.35–0.66).

**Fig 1:**
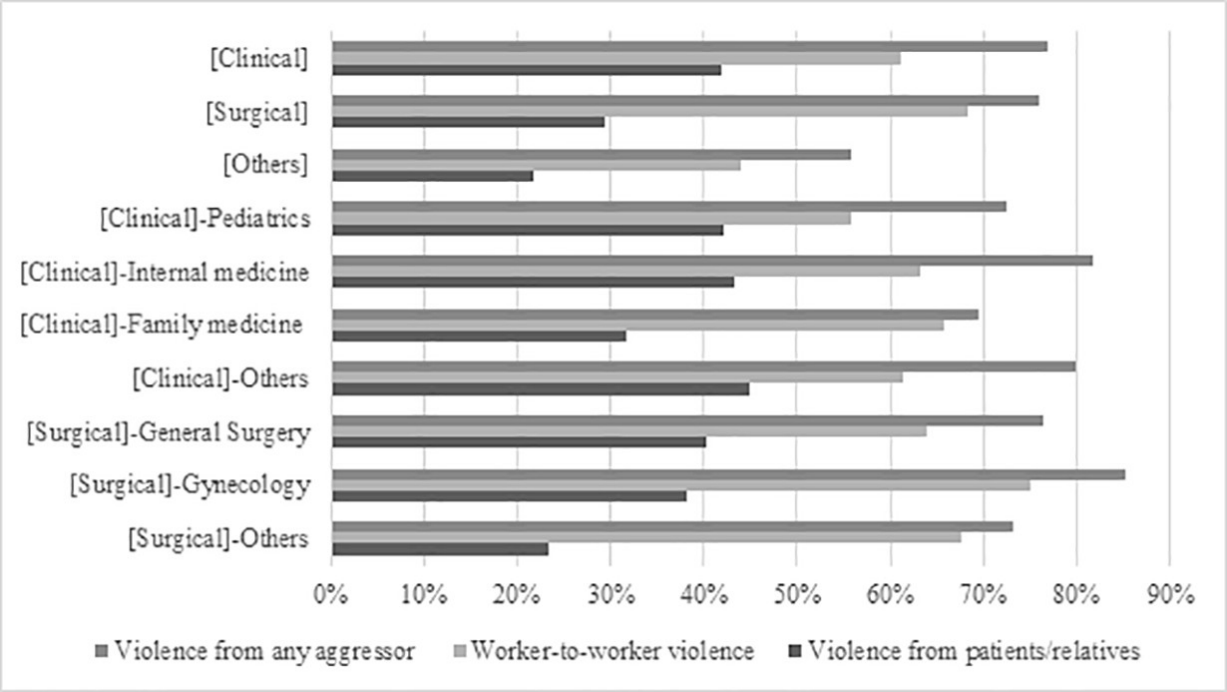
Prevalence of workplace violence according to specialty. Specialty group.

Prevalence of worker-to-worker violence was 61.2% in clinical residents, 68.3% in surgical residents, and 44.1% in residents of other specialties. Compared with clinical residents, this prevalence was higher in surgical residents (PR: 1.11, 95% CI: 1.01–1.23), but lower in the group of other specialties (PR: 0.76; 95% CI: 0.62–0.92) (Table 4 and Fig 1).

**Table 4:** Medical specialties associated with workplace violence according to type of aggressor (n = 1054).

Variables	Violence from patients/relatives PR (95% CI)*	Worker-to-worker violence PR (95% CI)*	Violence from any aggressor PR (95% CI)*
**Specialty group**			
Clinics (n = 521)	Ref	Ref	Ref
Surgical (n = 372)	**0.71 (0.59–0.87)**	**1.11 (1.01–1.23)**	0.99 (0.92–1.07)
Others (n = 161)	**0.48 (0.35–0.66)**	**0.76 (0.62–0.92)**	**0.75 (0.64–0.87)**
**Specialties**			
Paediatrics (n = 102)	Ref	Ref	Ref
Internal medicine (n = 60)	1.20 (0.84–1.72)	1.08 (0.83–1.42)	1.13 (0.95–1.35)
Family Medicine (n = 85)	0.95 (0.65–1.39)	1.21 (0.95–1.54)	1.01 (0.84–1.22)
Other clinical specialties (n = 274)	1.03 (0.78–1.36)	1.09 (0.88–1.34)	1.09 (0.94–1.25)
General surgery (n = 72)	1.00 (0.68–1.48)	1.11 (0.85–1.43)	1.05 (0.86–1.27)
Gynecology (n = 68)	1.03 (0.71–1.48)	**1.29 (1.03–1.62)**	**1.16 (0.99–1.36)**
Other surgical specialties (n = 232)	**0.60 (0.43–0.83)**	**1.22 (0.99–1.50)**	1.03 (0.89–1.20)
Others (n = 161)	**0.49 (0.33–0.72)**	0.83 (0.64–1.07)	**0.79 (0.65–0.95)**

*These regressions were adjusted by sex, age, university responsible of the residency, city where the residency is undertaken, migration, year of residency, health system of the institution and hours worked per day.

Bold numbers indicate p < 0.05

## Discussion

### Main findings

Of the 1054 medical residents evaluated, 73.4% reported having suffered some type of workplace violence, 34.4% reported violence from patients/relatives, and 61.1% reported worker-to-worker violence. Workplace violence was similar among clinical and surgical residents, and lower in residents of other specialties. However, compared to clinical residents, surgical residents reported lower rates of violence from patients/relatives, but higher rates of worker-to-worker violence. The group of other specialties reported a lower prevalence of both types of violence.

### Workplace violence

The prevalence of workplace violence was 73.4%, similar to that reported in studies in the United States [7, 8], Iran [12, 13], Turkey [14], Japan [15], Argentina [19], Mexico [29], and Venezuela [30], in which the prevalence ranged from 66% to 97%. In addition, we found that verbal violence was more frequent than physical violence (72.7% vs 16.3%), similar to other studies conducted in different populations of residents [3, 7, 8, 13–15, 19, 31]. It is important to note that both physical and verbal violence can have a negative impact on the resident's emotional, academic, and professional status [32].

We found that the main perpetrators of workplace violence were other workers of the institution, followed by patients/relatives. Similar results have been reported in other studies in Turkey [14], Iran [13], Japan [15], Uruguay [17], Argentina [19], Mexico [29], and Venezuela [30]. However, studies in the United States [7] and Mexico [16] found that aggressors were mainly patients/relatives. Additionally, consistent with other studies, we found that attending physicians and senior residents were the main aggressors in terms of verbal violence [3, 8], while physical violence is usually caused by patients/relatives [4, 33].

### Medical specialty associated to workplace violence

Our study evidence that surgical residency was associated with suffering more worker-to-worker violence, especially for gynecology residents. This finding is in accord with studies in Mexico [16] and Venezuela [30]. This is probably because surgical staff is continuously exposed to adverse events with medico-legal consequences [34–36]. In addition, surgery departments in several Peruvian hospitals are characterized by being hierarchical and authoritarian, where verbal violence is usual in the teaching process, similar to that described in Mexico [29].

Surgical residents had lower workplace violence from patients/relatives than clinical residents. This may be because surgical physicians have lower contact with patients/relatives, compared to clinical specialties. Among surgical residents, workplace violence from patients/relatives was lower for other surgical specialties (nor general surgery nor gynecology), probably because general surgery and gynecology have a higher number of patients and surgical procedures compared to the other surgical specialties, possibly resulting in a higher incidence of complications and surgical deaths with consequent patient/relatives dissatisfactions [3, 37]. In addition, it has been postulated that the conflicts between doctors and patients is an expected consequence of a greater availability of online health information along with a poor doctor-patient communication. Ergo, when the patients come with certain information about their disease and doctors cannot communicate adequately to them (about their condition, the disease process, the therapy, etc.), it leads to trouble and in extreme cases to violence [38].

Our results suggest that interventions to prevent worker-to-worker violence should be undertaken mainly for surgical residents. These interventions may include training in the detection and timely reporting of workplace violence [39–44]. Furthermore, *ad hoc* hospital and extra-hospital committees with the participation of residents representatives could be created [45].

The interventions to prevent violence from patients/relatives should be promoted mainly for clinical residents. These interventions may include ensuring the presence of security personnel during the interaction between residents and patients/relatives, especially with those who are under drugs or alcohol influence, or suffer mental disorders. [4, 7, 46, 47]. In addition, hospitals could impose harsher punishments for committing violence against health workers; this has shown to be an effective deterrent in Spain, where workplace violence rates have reduced [48]. Television campaigns showing that violence against health workers as an intolerable issue also have been successful in reducing violence in England [49] and South Africa [50].

The prevalence of violence from patients/relatives and worker-to-worker violence were lower among residents of other (non-clinical and non-surgical) specialties. This may be because many of these specialties, such as health management, radiology, laboratory, among others, have less contact with patients/relatives, which would reduce their risk of violence [51]. In addition, these specialties may have less workload and fewer hours worked per day [52, 53], which would reduce their exposure to episodes of workplace violence [54].

### Limitations and strengths

Some limitations of this study should be mentioned: 1) The workplace violence can be considered subjective, so the definition of an act as violence may vary according to the respondent, as suggested in previous studies [12, 14]. However, this variable was collected through questions based on definitions of the International Labor Office [27], which should have allowed a standardization of the concepts. 2) The sample size did not allow us to evaluate each specialty separately, and the specialties categorization (clinical, surgical, and others) may be placing very different specialties in the same group. 3) The study has a relatively low response rate, likely because it was a virtual voluntary survey, so the percentages of violence could be underestimated; thus, generalization should be done carefully. 4) In addition, residents of more distant hospitals, or those who work more daily hours, who in turn could have a higher prevalence of workplace violence, could be less prone to answer the survey, which may lead to an underestimate of workplace prevalence in our results.

However, the present study is one of the few performed in Latin America that assess the problem of workplace violence in medical residents. This study helps towards better the understanding of aggression towards doctors in the workplace and looks at the sources for this aggression. It has included many cities and institutions throughout Peru, and its results allow the identification of specialties with a higher violence prevalence according to perpetrator, which can be used to formulate preventive policies.

### Conclusion

Nearly three quarters of medical residents reported having suffered workplace violence sometime during the residency. Compared with clinical residents, surgical residents had lower rates of violence from patients/relatives, but higher rates of worker-to-worker violence, while residents from other (non-clinical, non-surgical) specialties had a lower prevalence both types of violence.

## Abbreviations

ENMERE: Peruvian Medical Residents National Survey
CONAREME: National Committee of Peruvian Medical Residency
MINSA: Ministry National of Health
ESSALUD: Social Security
ILO: International Labor Office
